# Supplementary figures and images for: Activity of biogenic silver nanoparticles in planktonic and biofilm-associated Corynebacterium pseudotuberculosis
Source: PeerJ. 2024 Feb 20;12:e16751. doi: 10.7717/peerj.16751 (PMC10885795; doi:10.7717/peerj.16751)

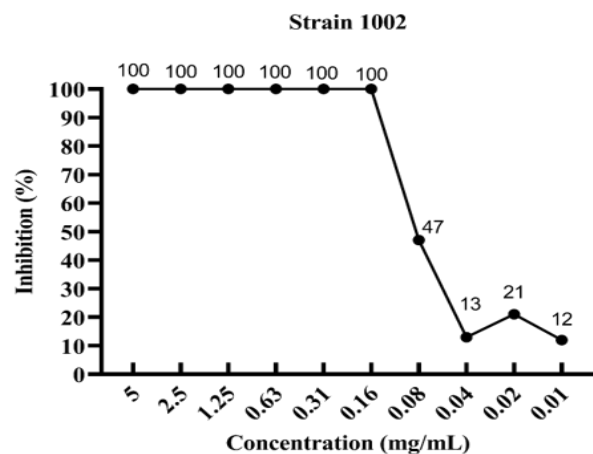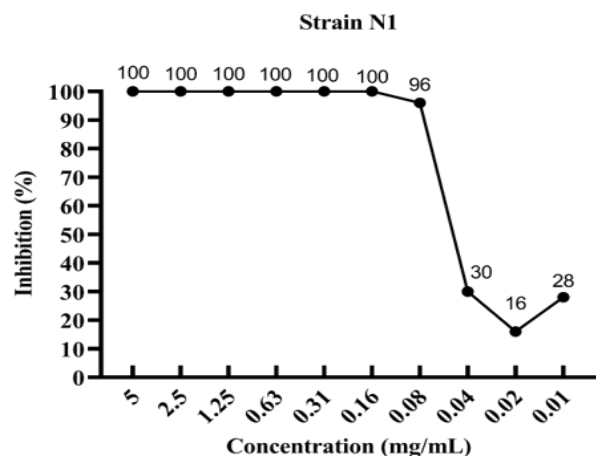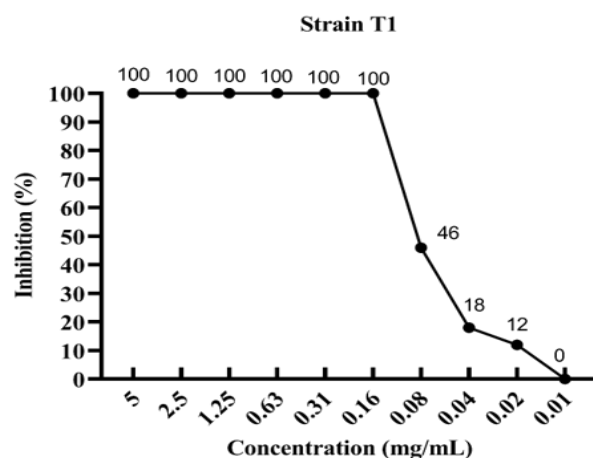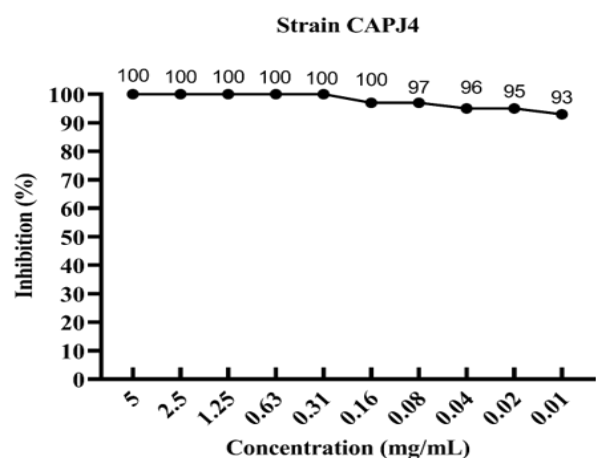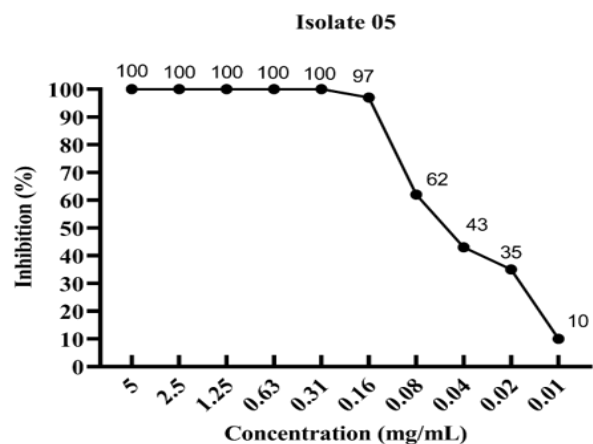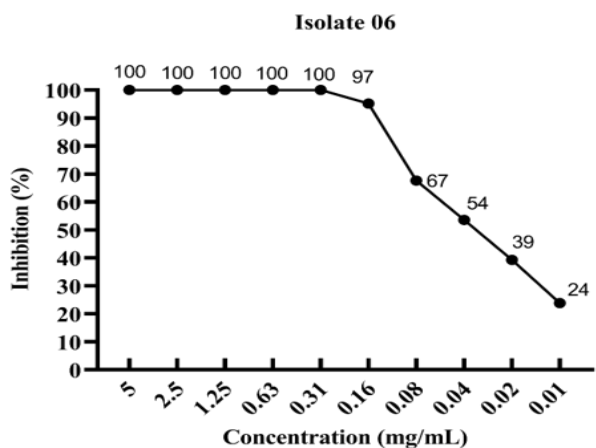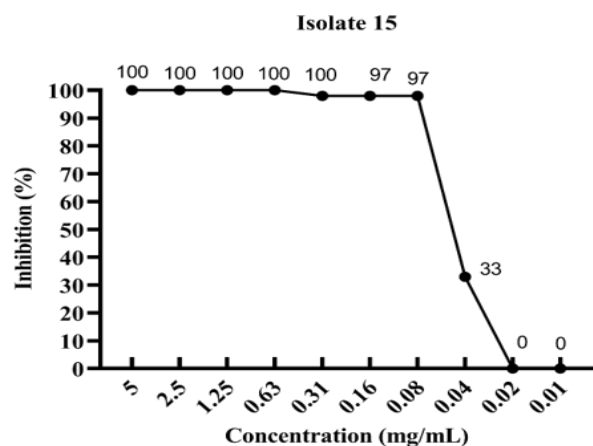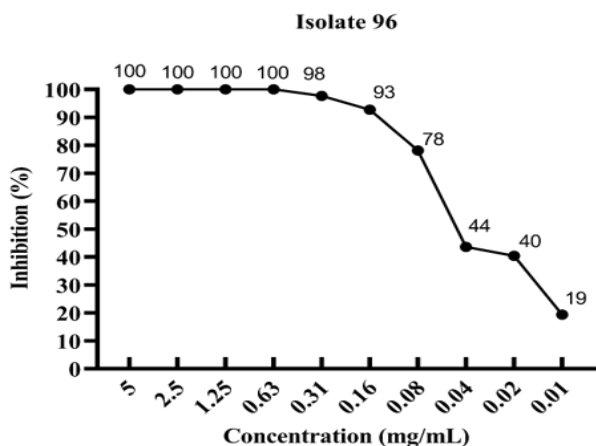

Supplement: Figure S1 — The results express the means of growth inhibition, in percentage, as shown by independent experiments. [file peerj-12-16751-s001.pdf]
